# Time to change? Present and prospects of hemorrhoidal classification

**DOI:** 10.3389/fmed.2023.1252468

**Published:** 2023-10-11

**Authors:** Ling Wang, Jiachun Ni, Changcheng Hou, Di Wu, Li Sun, Qiong Jiang, Zengjin Cai, Wenbin Fan

**Affiliations:** ^1^Chongqing College of Traditional Chinese Medicine, Chongqing, China; ^2^Chongqing Medical University, Chongqing, China; ^3^Department of Proctology, Yongchuan Hospital of Traditional Chinese Medicine, Chongqing Medical University, Chongqing, China; ^4^Department of Coloproctology, Yueyang Hospital of Integrated Traditional Chinese and Western Medicine, Shanghai University of Traditional Chinese Medicine, Shanghai, China

**Keywords:** hemorrhoids, classification, surgical procedures, surgical operative, review

## Abstract

As a common benign anal condition, the high incidence and recurrence of hemorrhoids pose challenges for both patients and doctors. The classification of hemorrhoids plays a crucial role in assessing, diagnosing, and treating the condition. By using appropriate classification and corresponding treatment strategies, we can achieve higher cure rates and lower recurrence rates of hemorrhoids. Since the introduction of the Miles classification in 1919, various classifications have been developed, which include objective classifications based on anatomical or instrumental assessment and subjective classifications based on symptoms and patient sensations. These classifications aim to accurately evaluate the condition. In this study, we discuss the evaluation values of each classification in terms of their advantages, disadvantages, treatment relevance, reproducibility, practicality, and assessment value. We also analyze the significant and essential factors, principles of use, and components of assessment indicators of hemorrhoidal classification. This study proposes several strategies to address the limitations of current hemorrhoidal assessment methods. All these will provide a reference for the development regarding the assessment and classification of hemorrhoids and clinical diagnosis and management of hemorrhoids.

## 1. Introduction

**Figure 1:**
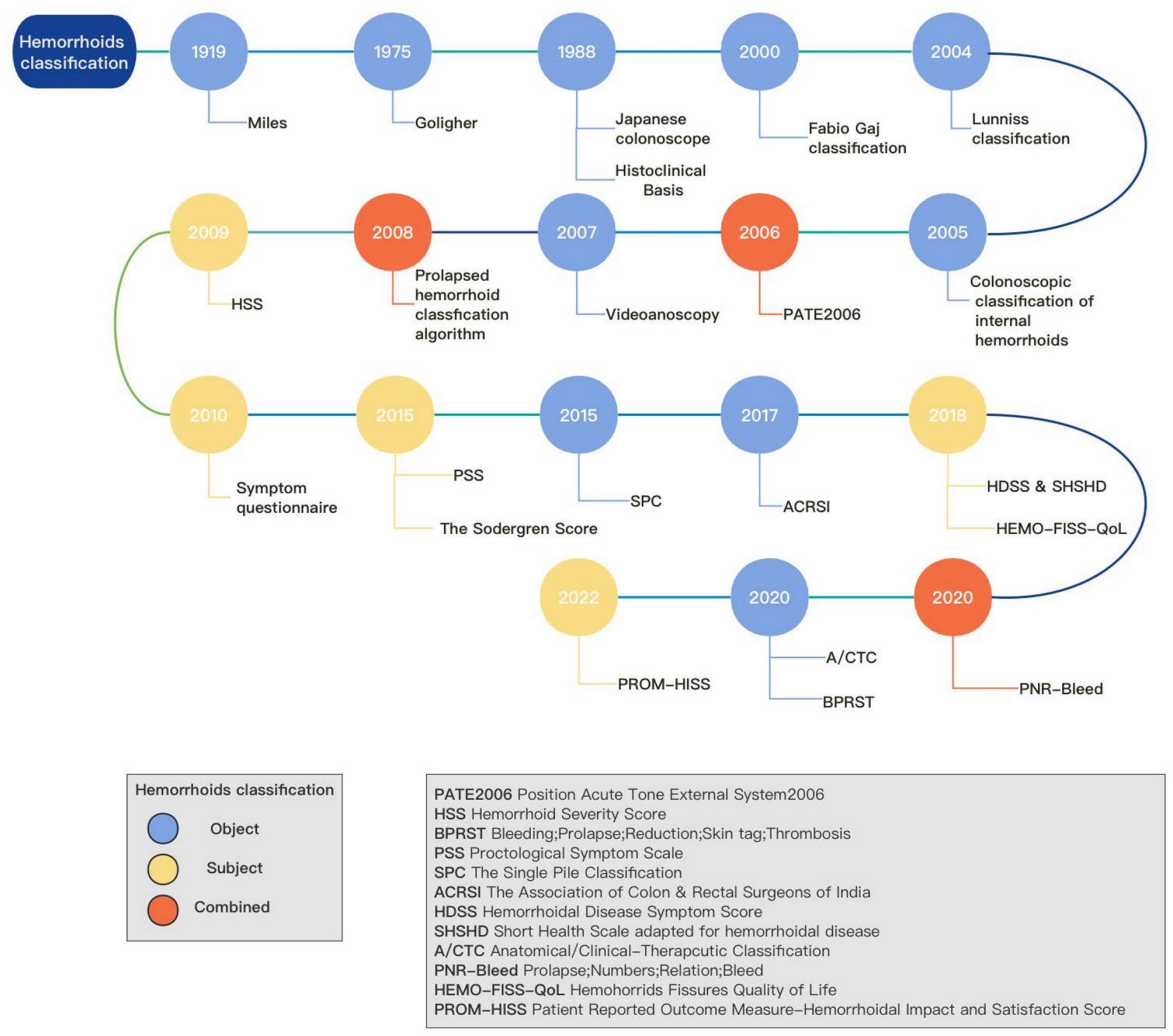
History of hemorrhoids classification.

Hemorrhoids are a prevalent condition among adults. According to a survey conducted in 2010, which focused on outpatient diagnoses of gastrointestinal, hepatic, and pancreatic diseases in the United States, hemorrhoids ranked as the third most common disease (1). Its symptoms include bleeding, pain, prolapse, and pruritus (2). The pathophysiology of hemorrhoids and the development of new treatment techniques and clinical management of treatment lack a consensus (3). The assessment and classification of hemorrhoids are essential for the treatment and management of this condition. Classification is significant in the medical field as it aids in identifying anatomical defects based on symptoms, which may require surgical correction. Additionally, it allows for the measurement of symptoms and complaints before and after treatment, enabling comparison. Moreover, it aids in developing a surgical strategy that is shared with the patient, taking into account not only anatomical corrections but also postoperative discomforts such as pain and disability. Hence, an effective tool should consider both the anatomical outcomes and the associated symptoms. Currently, the Goligher classification is widely used as the standard classification system (4), which classifies hemorrhoids based on internal hemorrhoidal prolapse. However, it is important to note that the Goligher classification has limitations and may not fully consider specific clinical conditions, such as circumferential prolapse and thrombosis (5). So far, many different classifications and treatments of hemorrhoids have been developed (6) (Figure 1). The one-sided classification has resulted in a lack of consistency in treatment choices made by physicians, which is detrimental to the overall treatment outcomes. This inconsistency may arise from the disparity between the objective and subjective aspects of the patient, further contributing to the heterogeneity observed in treatment approaches (7). However, most of the emerging methods are not widely used. To understand the reasons behind this, this article provides a comprehensive review of the development and formulation of hemorrhoid classification methods.

## 2. Hemorrhoidal classification

Hemorrhoids have been the subject of extensive research by scholars since the nineteenth century. Various classification methods for hemorrhoidal disease have been developed, considering factors such as pathophysiology, anatomy, and associated symptoms. The characteristics of hemorrhoids can be described objectively through signs such as the localization and morphology of the piles plexus and subjectively through symptoms and the perceived impact on quality of life (QoL). Most existing classification methods for hemorrhoids are based solely on objective or subjective characteristics, which have their own limitations and shortcomings. The Goligher classification remains the most widely used method. However, with advancements in our understanding of the pathophysiology and the development of new treatment techniques, there is no consensus on the clinical and therapeutic aspects of hemorrhoids. This lack of agreement has resulted in heterogeneity in the management of the disease (8). Various treatment options are available for managing modern hemorrhoids, and the selection of a suitable strategy should be based on individual patient characteristics and clinical factors. However, the diverse range of interventions can pose challenges when comparing their effectiveness.

### 2.1. Subjective hemorrhoidal classifications

#### 2.1.1. Hemorrhoid severity score

In 2009, P.-O. Nyström and his team presented the first questionnaire, named Hemorrhoid Severity Score (HSS), to assess the symptoms of patients with hemorrhoids in a study on whether stapled anopexy or diathermy excision of hemorrhoids could improve the patient's symptoms (9). The questionnaire consists of five questions about the frequency of hemorrhoid-related symptoms (pain, itching, bleeding, prolapse, anal discharge) and further assesses patients' pain and incontinence. Assessment from the patient's perspective provides a detailed understanding of the level of pain associated with mucosal anal prolapse. This questionnaire was proposed and used by many clinical studies (10–12). For the HSS to be reliably validated and to establish that the HSS is an appropriate scale to use to measure aspects of hemorrhoidal symptoms, a multicenter parallel-group randomized controlled trial was performed by Lee et al. (13). The HSS was used to assess a cohort of patients treated by rubber band ligation (RBL) or hemorrhoidal artery ligation (HAL) and also the Vaizey incontinence score to assess patients' incontinence using four methods (effect size, standardized response means (SRM), significance of change, and responsiveness statistic) to compare the responsiveness of the two questionnaires. The results suggest the responsiveness statistic with the Vaizey ranged from 0.23 to 0.38, while the responsiveness statistic with the HSS reached 1.02–1.45, indicating that the HSS was more responsive and sensitive to changes in patient health status, further supporting the validity of the HSS. A limitation of this trial, however, is that only quantitative data were obtained, emphasizing only the generalized results of the questionnaire and possibly ignoring a small number of specific groups.

#### 2.1.2. Symptom questionnaire

Giordano et al. employed a symptom questionnaire to evaluate two surgical treatments for stage II and III hemorrhoids. employed a symptom questionnaire to evaluate two surgical treatments for stage II and III hemorrhoids (14). The questionnaire encompassed various aspects such as bleeding, prolapse, manual reduction, impact on the patient, pain or discomfort, and QoL, considering the severity of hemorrhoidal symptoms. Postoperative pain was carefully assessed using the visual analog scale (VAS), and patient satisfaction with treatment was also examined. Although the questionnaire results were not directly correlated with the clinical trial outcomes, it facilitated the assessment of the patient's condition during the telephone follow-up. This questionnaire offers a novel perspective for future research.

#### 2.1.3. Proctological symptom scale

Matthias et al. worked on the development of a questionnaire proctological symptom scale (PSS) for assessing symptom severity in benign rectal disease (15). The study had 229 patients with the rectal disease and 133 patients without the rectal disease (control group) and compared whether the PSS could be partially or fully replaced by the widely used incontinence score and the constipation score (16, 17). The results of the trial were that the PSS was able to differentiate patients with rectal disease from non-rectal disease controls and, following the intervention, also differentiate treatment success from failure. Notably, failure of these interventions was detected early in the course of treatment (after the first treatment), which may imply that the PSS has some prognostic value.

#### 2.1.4. HEMO-FISS-QoL

Abramowitz et al. developed another questionnaire regarding the overall impact of hemorrhoids and anal fissures on the daily life of patients (18). The findings were that among patients with hemorrhoids and anal fissures, scores on the HEMO-FISS-QoL questionnaire increased with the severity of symptoms (pain, bleeding, and prolapse) and with the impact on daily life. This is the first known comprehensive psychological development questionnaire for hemorrhoids and anal fissures, and some researchers have found this study useful (19, 20). A limitation of this trial is that no survey assessment of patients before and after treatment was made, and clinical sensitivity was lacking.

#### 2.1.5. Hemorrhoidal disease symptom score

Roverik conducted a study to assess the validity, reliability, and responsiveness of patients' self-reported scores of pain, itching, bleeding, soiling, and prolapse symptoms (Hemorrhoidal Disease Symptom Score) (21). The HDSS was modified from the HSS by changing the score from one based on the patient's feelings in the last 2 weeks to 3 months and a change in the scale. In addition, the reliability and responsiveness of an instrument that measures health-related quality of life in patients with hemorrhoids (Short Health Scale HD) were also assessed. The results showed that the HDSS and SHS_HD_ were able to provide surgeons with a good overview of the symptoms experienced by patients and their impact on daily life and health. However, the validity of a classification should always be measured in the setting of the purpose for which it is being used and the population for which it is being validated (22). HDSS and SHS_HD_ are primarily used to assess symptoms. They can neither be used to diagnose hemorrhoid disease nor to determine prognosis alone.

#### 2.1.6. The Sodergren score

In 2015, the Sodergren score assessment tool was proposed by Pucher et al. to assess the severity of symptoms and their quality of life in patients with hemorrhoids and can be used to compare treatments, monitor the disease, and assist in decisions about surgery (23). The researchers asked 45 patients with hemorrhoids to complete a questionnaire, and the data were then analyzed by a statistical tool. The results showed that the Sodergren score provides a clearer picture of the subjective impact of the disease on the patient and is a valid tool for assessing the severity of the condition. Although it is effective for stratifying symptoms by severity, it is not an aid for diagnosis. In patients with complex mixed pathology, the diagnosis should still be considered in light of the actual condition. The limitation of this study is that the sample size of the trial was too small, and its reliability and responsiveness were not analyzed. Therefore, Sha et al. performed a study to assess the Sodergren score (24). This study was designed to see if there was a difference in the Sodergren score between patients who underwent surgical treatment and those who had successful internal hemorrhoid rubber band ligation (RBL) and to assess whether the score could provide therapeutic guidance for treatment. Unlike the HDSS, which focuses on the frequency of symptoms, the Sodergren score also assesses the frequency and severity of symptoms, which is important for clinical management. The frequency of symptoms is not indicative of the severity of the condition, and the two should be distinguished (25). The results showed a significant difference in scores between patients who underwent surgery and those who successfully underwent RBL for internal hemorrhoidal disease and recommended that patients with a pre-treatment Sodergren score of 6 or more should be considered for upfront surgery and those with a score below 6 should undergo RBL.

#### 2.1.7. PROM-HISS

In 2022, Kuiper et al. introduced a new evaluation tool for hemorrhoids called the Patient Reported Outcome Measure-Hemorrhoidal Impact and Satisfaction Score (PROM-HISS) (26). PROM-HISS was developed through a comprehensive review of existing literature and expert panel discussions. It assesses the impact of hemorrhoidal symptoms (such as blood loss, pain, prolapse, soiling, and itching) and HD on daily activities and measures patient satisfaction with treatment. The recall period for the items is set as ‘in the past week’. One of the strengths of PROM-HISS is its potential to support evidence-based surgical data and provide a quantitative and systematic understanding of patients' experiences with hemorrhoidal disease. Furthermore, PROM-HISS demonstrates good structural properties, internal consistency, and construct validity.

### 2.2. Objective hemorrhoidal classifications

#### 2.2.1. Classifications based on anatomy or symptoms

##### 2.2.1.1. Classifications in the early stage

In 1919, Miles published a review on internal hemorrhoids (27). The review proposed a classification of internal hemorrhoids into three stages based on anatomy and concluded that there were two indications for surgery for internal hemorrhoids: first, massive recurrent bleeding, and second, irreversible prolapse. In 1975, the Goligher classification was proposed, which classified the degree of prolapse of internal hemorrhoids into four grades (28). The Goligher classification lacks detailed anatomical descriptions, which hinders the expression of the variability and true severity of hemorrhoid disease. As a result, it often leads to inaccurate classification of most conditions. However, its simplicity has contributed to its widespread and sustained use over the decades. Several authors have developed alternative scoring systems to overcome these limitations. These elaborate classifications are not commonly used in clinical practice or internationally. The explanation for the difficulty in implementing other classifications compared to the Goligher system may be attributed to their relative complexity. Therefore, replacing the Goligher classification would pose significant challenges. In simple terms, it adds the additional classification of non-retrievable permanent prolapse to the classification of Miles. This classification is by far the most widely used in the world. The result is evaluated solely by the physician, who assesses it based on the patient's clinical history and examination. It is important to note that there may be variations in the interpretation of the findings among different physicians (29). Morgado PJ and others conducted a histological study in 1988 (30). They concluded that hemorrhoids are normal human tissue, any hemorrhoid without symptoms is not a disease, and it is unreasonable to classify them according to the size of normal human tissue. Symptoms should be used to classify hemorrhoids. Symptoms such as bleeding, thrombosis, and prolapse are clinical components of hemorrhoids and may occur independently of each other or together, independent of the size of the anal mass. Symptoms can also clearly distinguish acute conditions such as thrombosis, but thrombosis usually forms in external hemorrhoids and does not fall within the definition of internal hemorrhoids.

##### 2.2.1.2. Fabio gaj classification

A new classification was described by Gaj et al. (31). This classification is based on anatomy and symptoms and is graded according to the number of internal hemorrhoids and the extent of prolapse, the number of external hemorrhoids, and the occurrence of acute events (thrombosis and edema). A comparative clinical study of this classification and the Goligher classification, and a statistical study to compare them, found that the new classification was three times more powerful than the old one. In his description, the fourth stage of the old classification was incorrectly defined and lacked acute events, thus preventing doctors from correctly selecting whether a patient should undergo ambulatory surgery (AS) or one-day surgery (ODS).

##### 2.2.1.3. Lunniss classification

Lunniss et al. in 2004 proposed another new classification from a therapeutic point of view (32). The classification is based on morphology, by main symptoms (prolapse and bleeding) and accompanying symptoms (external hemorrhoids and pruritus, etc.), size of internal hemorrhoids, age of the patient, and recommendations for routine treatment.

##### 2.2.1.4. The single pile classification

In 2015, Elbetti et al. developed a new tool called the single pile classification (33). The authors combined it with the Goligher classification and analyzed 197 patients and found that the new classification was able to describe in more detail symptoms that were not included in the old classification. The lack of a detailed description of anatomy in the Goligher classification hindered the expression of hemorrhoidal variability and true severity.

##### 2.2.1.5. India classification

Niranjan et al. proposed a new concept of classification of hemorrhoids. proposed a new concept of classification of hemorrhoids (34). This classification is based on Goligher classification and divided into four categories (ABCD) based on different conditions such as the number of hemorrhoids, the proportion occupying the circumference of the anal canal, and whether there is thrombosis or gangrene. It aims to overcome the limitations of the Goligher classification by providing additional information on the management process and treatment of hemorrhoids, thus assisting in clinical diagnosis and treatment.

##### 2.2.1.6. Anatomical/Clinical–Therapeutic Classification (A/CTC)

Naldini et al. applied a new classification for hemorrhoids in 2020 called the anatomical/clinical–therapeutic classification (A/CTC), which concluded that the Goligher classification lacked quantification of prolapse, assessment of symptom type, and did not correlate with treatment modalities, and assessed whether the classification improves postoperative outcomes (35). This classification includes an assessment of the anatomical features of hemorrhoids (internal prolapsed hemorrhoids and external hemorrhoids), the type of symptoms (bleeding, overflow, edema, etc.), the frequency of symptoms, and a list of complications to be aware of and contraindications to treatment modalities, which can help to avoid the risks of surgery. However, this assessment classification method is cumbersome to assess and therefore not very practical. The authors investigated 381 patients with symptomatic hemorrhoids who underwent surgical treatment and were followed up for an average of up to 30 months after surgery. The results showed satisfactory postoperative outcomes for all hemorrhoid disease procedures. These good results may be due to the patient's choice of the correct treatment and the appropriate indications for surgery.

##### 2.2.1.7. BPRST

In 2020, a classification called BPRST was also proposed by Sobrado júnior et al. (36). The authors concluded that proposing a new classification model for hemorrhoids should consider the disease holistically, addressing not only prolapse but also other equally important symptoms such as external hemorrhoids and thrombosis. As with other classifications, this author also assessed the association between the Goligher classification and our proposed BPRST classification. He analyzed and compared the admission Goligher classification and the treated BPRST classification in 149 patients with symptomatic hemorrhoids and assessed the correlation with the treatment they used. The results showed that the BPRST staging was more accurate than the Goligher classification, with less variation in the treatments used by patients in the sample.

#### 2.2.2. Classification based on instrument check

##### 2.2.2.1. Retroflexed fiberoptic colonoscope

In 1998, Sadahiro et al. proposed a classification for the use of a retroflexed fiberoptic colonoscope for the assessment of internal hemorrhoids (37). The authors investigated 531 patients with complaints of rectal or anal symptoms, first using an anoscope to look at the left side of the anal region and then observing through an intrarectally retroflexed fiberoptic colonoscope. The results of the study showed that red signs were strongly associated with bleeding, and retroflexing the colonoscope intrarectally helped to identify findings in the anal canal associated with hemorrhage and prolapse. The authors concluded that the advantage of this classification is that the retroflexed fiberoptic colonoscope provides a clearer and more extensive view than the anoscope and allows a better assessment of the color and presentation of the anal canal surface. In 2005, this classification was refined by Fukuda et al. who used colonoscopy to assess internal hemorrhoids (38) and assessed 104 patients with symptomatic internal hemorrhoids using posterior curvature and anterior view angles of the colonoscope. The trial focused on comparing the patients' changes in colonoscopic observations before and after treatment and found that all showed improvement. The study showed that the new endoscopic classification of internal hemorrhoids proved to be closely associated with symptoms, particularly bleeding, and was therefore useful in assessing the effectiveness of treatment. The authors concluded that the classification has the advantage of assessing fewer items than that proposed by Sasahiro, which is not only simpler but also reduces observer variability and improves reproducibility.

##### 2.2.2.2. Videoanoscopy

Harish et al. developed another method of assessing hemorrhoids using videoanoscopy and conducted a study designed to make a comparison with the retroflexed fiberoptic colonoscope (39). The investigators screened 544 patients who were symptomatic in the anorectum only and then assessed the patients using both methods. The videoscope is a sigmoidoscope or colonoscope with the tip inserted into the inner lumen of the anoscope and advanced to its tip. The items assessed are the size and number of hemorrhoids and the red corpuscular sign (RCS). The results showed that the number of subjects with hemorrhoids detected by videoanoscopy was significantly higher compared to a retroflexed fiberoptic colonoscope. Hemorrhoids are the main cause of acute or recurrent rectal bleeding; however, it cannot be ruled out whether the bleeding is due to other lesions, such as gastrointestinal malignancy, when assessing the patient's condition by the everyday physician (40). Although anoscopy is highly sensitive in identifying pathologies such as internal hemorrhoids, the results of anoscopy do not allow clinicians to exclude a proximal source of gastrointestinal bleeding. This is why videoscopy allows further investigation of the cause of bleeding and can be done without missing a diagnosis.

### 2.3. Combined hemorrhoidal classifications

#### 2.3.1. PATE2006

In 2006, a classification called the Position Acute Tone External (PATE) 2006 classification was developed (41). This classification aims to assess patients with hemorrhoids using objective indicators and QoL and uses a scoring system where the sum of the scores adds up to the outcome. The authors used the PATE 2006 classification on 500 patients before treatment and then assessed using the scoring system at postoperative follow-up. The results of the trial showed a direct correlation between the numerical score of the classification and the severity of the disease.

#### 2.3.2. Prolapsed hemorrhoid classification algorithm

Gerjy et al. proposed a prolapsed hemorrhoid arithmetic score method in 2008 (42). The algorithm was first divided into two categories based on patient self-report (need for manual retraction of the hemorrhoid nucleus), and denied patients were then subdivided based on preoperative and postoperative proctoscopic examination (presence of prolapse) by the surgeon at 3–6 months follow-up. Patients with external hemorrhoids will be further classified based on the number and proportion of external hemorrhoids occupying the perianal area. The results show that this anatomically based approach reliably typifies prolapsed hemorrhoids and also defines recurrent hemorrhoids.

#### 2.3.3. PNR-bleed

In 2020, Khan et al. conducted a study on a new classification of hemorrhoids called the “PNR-Bleed” classification (43). This classification included the degree of hemorrhoidal prolapse, the number of primary hemorrhoidal columns involved, the relation of the hemorrhoidal tissue to the dentate line, and the amount of bleeding. In addition to assessing the above symptom-based or anatomical items, the Hemorrhoid Severity Score (HSS) was also added. The authors believe that this classification allows comparison of treatment outcomes, relapse rates, and various complications of various treatment options. However, the HSS cannot be used as a basis for determining the severity of hemorrhoids in different patients.

## 3. Value analysis

### 3.1. Analysis of the assessment value and limitations of hemorrhoidal classifications

#### 3.1.1. Essential elements of hemorrhoidal classifications

1. Objectivity: The classification should accurately reflect the realistic portrayal of hemorrhoids and aim to minimize discrepancies among evaluators, thus ensuring accurate assessment and treatment.
2. Subjectivity: The classification should include the patient's subjective criteria to better assess the progress from the patient's perspective. Sometimes, the patient's own feelings may not align with their verbal expression due to various factors. However, if there are any doubts about the objective signs, the patient's subjective feelings can be used as a reference to evaluate the condition, such as itching, anal swelling, and other accompanying symptoms. Another approach to measuring the severity of hemorrhoids is through patient-reported outcome measures (PROMs). PROMs are increasingly important in clinical trials as they are patient centered and provide a standardized measure of disease outcomes (44).
3. Practicality: The classification method is simple and practical, and it has a positive effect on the evaluation of hemorrhoids.
4. Correlation with treatment modality: The classification of hemorrhoid assessment is essential to ensure the suitability of the treatment method. Correlating the assessment with the treatment modality is highly advantageous in creating personalized treatment plans for patients and evaluating the effectiveness of the treatment.
5. Reproducibility: The consistency of assessment results among different assessors, healthcare institutions, regions, and even countries for the same group of patients assessed using the same assessment method. This can be achieved through single-center or multicenter prospective or retrospective studies.

#### 3.1.2. Value analysis and limitations

Tables 1–3 present the strengths and weaknesses of each hemorrhoid assessment classification and their corresponding assessment values. The HDSS and SHSHD, the Sodergren score, and PROM-HISS are recommended for subjective hemorrhoidal classifications, while the New Indian hemorrhoid Classification, BPRST, SPC, and Prolapsed hemorrhoid Assessment Process are recommended for objective hemorrhoidal classifications. Although these methods are valuable, there is still room for improvement. Some limitations include the complexity of the operation, lack of evidence-based medicine, lack of correlation with treatment modalities, and absence of satisfaction measurement. While some assessment classifications have been examined in prospective clinical studies, they fail to compare the variability of results between clinicians at different centers and do not address the potential risk of bias among assessors. It is crucial to ensure the consistency of assessment results across assessors and centers to enhance the clinical reliability of hemorrhoid assessment classification. It is important to note that the use of the A/CTC classification is not recommended. We should avoid employing classification and measurement tools that rely on unclear or fanciful pathophysiological assumptions. For instance, certain theories propose that recto-anal intussusception is the underlying cause of hemorrhoids, or that correcting vascular hyper-flow through dearterialization is effective. These theories lack substantial evidence and should not be endorsed. This study, however, has some limitations, as discussed in this article. It is important to note that this study is not a systematic review, it is not registered, and the articles included in this study are all in English.

**Table 1:** Subjective hemorrhoidal disease classification.

**Classification**	**Advantage**	**Disadvantage**	**Treatment relevance**	**Reproducibility**	**Practicality**	**Assessment value**
HSS	The first subjective survey question to be asked paper; good responsiveness;	No satisfaction assessment; not associated with treatment modality		⋆	⋆	⋆⋆
Symptom questionnaire	Comprehensive	Not associated with treatment modality; lack of evidence-based medical evidence			⋆	⋆
PSS	Suitable for all patients with rectal disease	Not associated with treatment modality;		⋆	⋆	⋆⋆
HEMO-FISS-Qol	The only questionnaire that deals with sexuality; for patients with hemorrhoids and anal fissures	No reactivity testing; not associated with treatment modality; no comparison of scores between patients with outpatient and surgically removed hemorrhoids		⋆	⋆	⋆⋆
HDSS & SHS_HD_	Combining symptoms with patient satisfaction; reliability and validity are complete and reliable	Not associated with treatment modality		⋆	⋆	⋆⋆
The Sodergren score	Ability to assess the severity and frequency of hemorrhoid symptoms	No satisfaction assessment	⋆	⋆	⋆	⋆⋆⋆
PROM-HISS	Good structural properties, internal consistency, and construct validity; comprehensive	Not associated with treatment modality		⋆	⋆	⋆⋆

If the hemorrhoidal disease classification fully satisfies the corresponding necessary factors ⋆; if the hemorrhoidal disease classification partially satisfies the corresponding necessary factors ✰; if the hemorrhoidal disease classification does not satisfy the corresponding necessary factors, no indication is given. HSS, Hemorrhoid Severity Score; PSS, Proctological Symptom Scale; HEMO—FISS-QoL, Hemohorrids Fissures Quality of Life; HDSS, Hemorrhoidal Disease Symptom Score; SHS_HD_, Short Health Scale adapted for hemorrhoidal disease; PROM-HISS, Patient Reported Outcome Measure-Hemorrhoidal Impact and Satisfaction Score.

**Table 2:** Objective hemorrhoidal disease classification.

**Classification**	**Advantage**	**Disadvantage**	**Treatment relevance**	**Reproducibility**	**Practicality**	**Assessment value**
Miles	Associated with treatment modality	Lack of evidence-based medical evidence; lack of classification of internal and external hemorrhoids	✰		⋆	✰⋆
Goligher classification	Wide range of application	Lack of bleeding and classification of internal and external hemorrhoids; not associated with treatment modality; lack of evidence-based medical evidence			⋆	⋆
Histoclinical basis	Novel; consideration of complications	Lack of classification of internal and external hemorrhoids; not associated with treatment modality; lack of evidence-based medical evidence			⋆	⋆
Lunniss classification	Comprehensive; refine the classification of non-prolapsed hemorrhoids	Complicated; lack of evidence-based medical evidence	⋆			⋆
SPC	Novel; focusing on the single piles; describe the anatomy in detail	Not associated with treatment modality		⋆	⋆	⋆⋆
India hemorrhoid classification	Subdivide of the number, circumference and thrombosed hemorrhoids	Lack of bleeding and classification of internal and external hemorrhoids; lack of evidence-based medical evidence	⋆		⋆	⋆⋆
A/CTC	Quantifying prolapse; concern about complications and contraindications to treatment modalities	Complicated	⋆	⋆		⋆⋆
BPRST	Novel; comprehensive	Complicated; ambiguous treatment modalities	⋆	⋆		⋆⋆
Japanese colonoscope classification	More objective assessment of bleeding	Not for general use; rated by the guide rated as non-standard; not associated with treatment modality		⋆		⋆
Colonoscopic classification of internal hemorrhoids	Reduces observer variability	Not for general use; not associated with treatment modality		⋆		⋆
Videoanoscopy	Wide view; high sensitivity; unexplained gastrointestinal bleeding can be ruled out	Not for general use; not associated with treatment modality		⋆	✰	⋆✰
Fabio Gaj classification	Classification according to the number of hemorrhoids and complications	Not associated with treatment modality		⋆	⋆	⋆⋆

If the hemorrhoidal disease classification fully satisfies the corresponding necessary factors ⋆; if the hemorrhoidal disease classification partially satisfies the corresponding necessary factors ✰; if the hemorrhoidal disease classification does not satisfy the corresponding necessary factors, no indication is given. SPC, The Single Pile Classification; A/CTC, Anatomical/Clinical-Therapcutic Classification; BPRST, Bleeding;Prolapse; Reduction; Skin tag; Thrombosis.

**Table 3:** Combined hemorrhoidal disease classification.

**Classification**	**Advantages**	**Disadvantages**	**Treatment relevance**	**Reproducibility**	**Practicality**	**Assessment value**
PATE2006	Comprehensive; novel	Not associated with treatment modality		⋆	⋆	⋆⋆
Prolapsed hemorrhoid classification algorithm	Clear processes; easy to operate	Not associated with treatment modality		⋆	⋆	⋆⋆
PNR-bleed	Comprehensive; subdivision IV hemorrhoids; quantifying bleeding symptoms	Not associated with treatment modality; Lack of evidence-based medical evidence			⋆	⋆

If the hemorrhoidal disease classification fully satisfies the corresponding necessary factors ⋆; if the hemorrhoidal disease classification partially satisfies the corresponding necessary factors ✰; if the hemorrhoidal disease classification does not satisfy the corresponding necessary factors, no indication is given. PATE2006, Position Acute Tone External System2006; PNR-Bleed, Prolapse; Numbers; Relation; Bleed.

#### 3.1.3. Novel strategies

To address the limitations, this article proposes the following optimization strategies for reference: (1) “Synonymous” substitution: This involves replacing assessment items that are subject to doubt in terms of subjectivity or objectivity with objective assessment items that have a causal link. For example, terms such as ‘fecal emission’ or ‘overflow’ can be replaced with ‘anal sphincter tone’. (2) Introduce computer-aided diagnosis (CAD): CAD refers to the analysis and modeling of patient data and images using computer-related technology. It aims to assist doctors in diagnosing patients and selecting appropriate treatment plans (45). Currently, computer-aided detection (CAD) has been extensively employed in the screening of breast cancer, lung cancer, and colorectal cancer (46–48). According to a recent study, the utilization of submucosal linear enhancement for computed tomography in patients with internal hemorrhoids has been found to enhance the detection rate of internal hemorrhoids that are at a high risk of bleeding (49). In future, the assessment and classification of hemorrhoids can be combined with computer-aided diagnosis (CAD) to simplify the assessment steps, improve efficiency, and enhance the usefulness of the assessment and classification. (3) Additionally, incorporating a subjective Patient-Reported Outcome Measures (PROM) questionnaire can provide a comprehensive understanding and assessment of the patient's condition from multiple perspectives. (4) The programs included in the new classification should be based on systematic literature reviews and Delphi consensus studies involving stakeholders. They should also encompass all aspects of the physical examination (50).

### 3.2. Clinical application recommendations

The pathogenesis of hemorrhoids is currently unknown. It is important to note that the objective scoring system determines the surgical strategy based on anatomical or symptom, while the subjective scoring system measures the clinical impact. Therefore, the classification of hemorrhoid assessment should be based on the patient's symptoms and complaints, following the principle of symptom/complaints orientation and allowing for flexibility.

#### 3.2.1. Conservative treatment or surgery

The choice between conservative and surgical treatment is a crucial decision that clinicians must make when treating patients with hemorrhoid disease. To aid in this decision-making process, clinicians can rely on the classification of hemorrhoid assessment, which provides valuable guidance: (1) The preferred assessment process for prolapsed hemorrhoids is the Goligher classification or the Prolapsed Hemorrhoid Assessment Process. The Goligher classification is represented by a flow chart, which can be used for clarity. Other assessment classifications, although cumbersome, can be used as deemed appropriate. (2) PNR-bleeding classification is primarily used for patients experiencing bleeding symptoms. It focuses on evaluating the quantity and frequency of bleeding and provides a detailed classification. In contrast, other assessment classification methods may have a more general approach or lack specific bleeding assessment items, allowing for flexible selection based on the specific situation.

#### 3.2.2. Choice of surgical procedure

(1) For prolapsed mainly: The New Indian Classification of hemorrhoids is the recommended classification system. It is derived from the Goligher classification, which evaluates the quantity and size of piles and provides a list of appropriate treatment options. This classification system is preferred due to its simplicity and minimal additional requirements. (2) For bleeding mainly: The PNR-bleeding classification is the preferred method, followed by the Japanese classification of internal hemorrhoid colonoscopy, which provides a more detailed assessment of bleeding symptoms. For patients with severe bleeding, it is recommended to consider sclerotherapy or transanal hemorrhoid artery ligation. If EBL treatment is being considered, the assessment can be done using the Japanese Classification of Internal Hemorrhoid Colonoscopy. (3) The use of SPC in surgical applications has proven to be highly effective. SPC involves a detailed description of the anatomy of each pathological pile, which enables close monitoring of the outcomes of medical or surgical treatments. This concept has led to the development of tailored surgeries, where the most suitable technique is employed for each specific pile, even within the same patient.

### 3.3. Future perspective

A validated and replicable assessment tool is essential for planning the ideal treatment strategy. The Core Outcome Set (COS) developed by van Tol includes the most commonly reported areas in studies on HD, such as pathophysiological presentation (including symptoms, complications, and recurrence rates) and patient satisfaction (51). According to the literature, the most common symptoms reported were pain (91%), blood loss (94%), and prolapse (71%). Among patients, pain and blood loss were found to be the two most common symptoms. The development of COS involved a content validity study, which included patient and expert feedback to establish relevance and comprehensiveness (52). This study will contribute to future research on hemorrhoid disease as it addresses the lack of formal content validity studies in subjective symptom-based classification. The proposed core set of outcomes can be beneficial for the further development of PROMS, not only in informing treatment decisions but also in evaluating treatment success and patient satisfaction (53). It is widely recognized that there can be a significant difference in the perception of treatment success between healthcare professionals and patients. This is because doctors primarily focus on observing the disease itself, while patients primarily experience the symptoms. The contrasting perspectives of healthcare professionals and patients can often lead to varying opinions regarding treatment outcomes (54).

Hemorrhoidal disease should be considered a progressive pathology as it can be influenced by other physiopathological conditions such as constipation, pelvic floor dysfunction, high anal resting tone, obstructed defecation, and childbirth. Therefore, it is important to take into account all comorbidities when defining hemorrhoidal pathology. In a benign scenario, quality of life serves as a significant indicator of the outcome. To ensure a more consistent approach to the treatment of HD and facilitate a uniform and standardized comparison of outcomes in future trials and prospective studies, the development of a new classification system is necessary. To classify HD, three main factors need to be defined: (1) the physiopathology of hemorrhoids; (2) the most common types of major symptoms; and (3) independent patient-specific characteristics such as underlying disease, gender, and age group. Additionally, the patient's subjective perception of the disease should be considered to better understand outcomes and provide prognostic care. It is crucial to translate the developed questionnaire into multiple languages and conduct cross-cultural validation, including cognitive interviews, with different patient groups. The validation of the new hemorrhoid classification should be done through an international multicenter trial, facilitated by one or more scientific societies of coloproctology. By widely implementing the new classification and guidelines, colorectal specialists and their national and international societies can improve the consistency of behavior, comparability of results, and ultimately enhance patient satisfaction.

## 4. Conclusion

Hemorrhoids are a common anorectal disease, with high incidence and recurrence rates, causing stress for both patients and doctors. Clinicians face the challenge of objectively and effectively assessing hemorrhoids and selecting appropriate treatment techniques. In recent years, various classification methods have been proposed by different scholars; however, no consensus has been reached. Some existing classifications still have issues, such as poor practicability and lack of correlation with treatment methods. These problems could be addressed in future by adapting the classifications or exploring new assessment classifications for hemorrhoids combined with computer-aided design (CAD) to simplify the assessment process and improve efficiency. It is important to note that most of the assessment classifications have not undergone further validation through clinical trials, which is a necessary step in establishing a new classification (55). The decision to change the classification system should not be made until a more practical classification is developed. However, some scholars argue for the need to update the classification system (29). The future course of action in clinical practice remains uncertain and should be observed. In future, both single-center and multicenter clinical trials can be conducted gradually to verify the clinical reliability and validity of these therapies. This will help in reaching a consensus and disseminating the findings. The treatment options for hemorrhoidal patients have improved, but there is still room for further advancements.

## Author contributions

LW and JN wrote the paper and outlined this manuscript. CH, DW, LS, QJ, and ZC were responsible for the idea of the article and accessed to information. WF provided a detailed guidance throughout the article. All the authors read and approved the final manuscript.
